# Enhanced Recovery after Surgery: History, Key Advancements and Developments in Transplant Surgery

**DOI:** 10.3390/jcm10081634

**Published:** 2021-04-12

**Authors:** Henry John Golder, Vassilios Papalois

**Affiliations:** 1 School of Medicine, Imperial College London, London SW7 2AZ, UK; 2 Department of Surgery, Imperial College London, London W12 0HS, UK; v.papalois@imperial.ac.uk

**Keywords:** enhanced recovery, ERAS, fast-track, transplantation

## Abstract

Enhanced recovery after surgery (ERAS) aims to improve patient outcomes by controlling specific aspects of perioperative care. The concept was introduced in 1997 by Henrik Kehlet, who suggested that while minor changes in perioperative practise have no significant impact alone, incorporating multiple changes could drastically improve outcomes. Since 1997, significant advancements have been made through the foundation of the ERAS Society, responsible for creating consensus guidelines on the implementation of enhanced recovery pathways. ERAS reduces length of stay by an average of 2.35 days and healthcare costs by $639.06 per patient, as identified in a 2020 meta-analysis of ERAS across multiple surgical subspecialties. Carbohydrate loading, bowel preparation and patient education in the pre-operative phase, goal-directed fluid therapy in the intra-operative phase, and early mobilisation and enteral nutrition in the post-operative phase are some of the interventions that are commonly implemented in ERAS protocols. While many specialties have been quick to incorporate ERAS, uptake has been slow in the transplantation field, leading to a scarcity of literature. Recent studies reported a 47% reduction in length of hospital stay (LOS) in liver transplantation patients treated with ERAS, while progress in kidney transplantation focuses on pain management and its incorporation into enhanced recovery protocols.

## 1. Introduction

Enhanced recovery after surgery (ERAS) programs aim to optimise pre-, intra- and post-operative care in order to improve the quality and speed of recovery in surgical patients. ERAS protocols are complex, requiring organised care from a multidisciplinary team to ensure strong patient outcomes and provide an elevated level of care. Enhanced recovery pathways reduce length of stay (LOS) by an average of 2.35 days and total cost by an average of $639.06 in comparison with conventional perioperative procedures, according to a 2020 meta-analysis of ERAS across multiple surgeries and surgical specialties [1]. Despite concerns, ERAS does not increase morbidity, mortality or readmission rates [2], having even been shown to decrease 30-day mortality rates following orthopedic surgery [1]. Since its beginnings as ‘fast-track’ surgery in 1997 [3], ERAS has been researched across a broad range of surgical subspecialties, from orthopedics to transplant surgery [4]. The ERAS Study Group (now the ERAS Society) formed in 2001 to develop consensus guidelines for perioperative care using the best available evidence [4], to encourage further research and to facilitate the discussion of enhanced recovery on an international scale.

Rapid advancements have been made in many areas of surgery, with some interventions having been accepted as beneficial across most, if not all fields. Mainstays of pre-operative care include patient education and counselling [5], while decisions regarding the use of mechanical bowel preparation, for example, remain controversial [6]. Recent years have seen major progression in intra-operative fluid management, with landmark studies beginning to suggest a shift away from conservative fluid administration to more specific goal-directed fluid therapy [7]. Finally, in post-operative care there is increasing evidence for early mobilization and enteral feeding of patients, owing to reductions in complications and LOS [8,9].

Despite marked progress in many subspecialties, the acceptance of ERAS has been slow in the field of solid-organ transplantation. Thus, there is limited existing literature in this area. Changes to USA organ allocation laws, which prioritise patients on the basis of pre-operative health, may disincentivise pre-surgical optimisation, an important aspect of many ERAS pathways [10]. Among other factors, this has played a key role in slowing the development in ERAS in liver transplantation (LT), though novel research is encouraging, as experts seek ways to optimise surgical pathways while working in accordance with updated regulations [11]. Recent years have also seen the first use of ERAS protocols in kidney transplants. Following successful feasibility trials in 2016 [12], research is currently focused on pain control and reduction in opioid requirements to improve patients’ experience and LOS [13]. Pregabalin-ketorolac regimens have shown positive results, and further studies are ongoing into the use of non-opiate analgesia [13].

This review aims to explore the history of enhanced recovery, from its inception as ‘fast track’ surgery to its use today. It also aims to explore the key advancements in ERAS to date by looking at landmark papers in perioperative care and to take a focused look into the use of ERAS protocols in transplant surgery.

## 2. History of Enhanced Recovery after Surgery

The concept of enhanced recovery after surgery (formerly ‘fast track surgery’) was introduced by Danish surgeon Professor Henrik Kehlet in 1997 [3]. Kehlet suggested that ‘while no single technique or drug regimen has been shown to eliminate postoperative morbidity and mortality, multimodal interventions may lead to a major reduction’ and went on to suggest pre-, intra- and post-operative surgical risk factors that may be addressed through coordinated perioperative protocols [3]. Following this, Kehlet published a trial that reported a mean post-operative hospital stay of 48 h after elective sigmoid resection [14]. Though this study included just 16 patients, fast-track surgery reduced post-surgical hospitalisation by 3–8 days [14].

In 2001 came the formation of the Enhanced Recovery After Surgery (ERAS) Study Group—a group of six surgeons assembled by Professors Ken Fearon and Ollie Ljungqvist and including Professor Kehlet, which aimed to produce and interpret the best available evidence to fine-tune fast track surgery [15,16]. Operating on the notion that ‘there was a great discrepancy between the actual practices and what was already known to be best practice’ [4], the ERAS Study Group published a review of the patterns of perioperative care in five northern European countries, concluding that colorectal perioperative protocols were neither standardized nor evidence based [17]. This was corroborated by a larger trial (*n* = 46,539), which found that crude mortality from cardiac surgery varied from 1.2% to 21.5% across Europe [18].

Maintaining its focus on colorectal surgery, the ERAS Study Group published what are now seen as the first consensus guidelines for perioperative care, conducting a 2005 review of enhanced recovery protocols in colorectal resections [19]. Enhanced recovery was becoming better researched, and its efficacy in colorectal surgery was consolidated in a 2010 meta-analysis of six randomized controlled trials, which reported that ERAS reduced length of hospital stay by 2.5 days and significantly reduced complication rates [20]. Following this, the Dutch Institute for Health Care Improvement conducted a trial to assess the ease of implementing ERAS protocols on a wider scale [21]. They enrolled 33 hospitals in the study and trained staff in a standardized ERAS protocols for elective colonic surgery, reporting significantly decreased LOS associated with early mobilisation and discontinuation of IV fluids as well as post-operative laxative administration [21]. While successful, the study did identify that adherence to the ERAS protocol fell to just 56% (calculated as mean average adherence to all post-operative aspects of the protocol) in the post-operative phase, having been 80% pre-operatively and 92% intra-operatively [21]. Studies have since highlighted the correlation between compliance and efficacy of ERAS protocols in both the short and long term [22,23].

In 2010, the ERAS Study Group registered as a new non-profit organization in Sweden under the title ‘the ERAS Society’, with the hope of evolving to reach other countries and create an international collaborative effort to improve perioperative protocols. Since its inception, the ERAS Society has published continuous research and guidance, held multiple symposia, and taken a leading role in the expansion of ERAS protocols to many new surgical subspecialties. It has also created an interactive audit system to help hospitals comply with ERAS protocols, making it easier for new hospitals to begin improving perioperative care. The main events in the history of ERAS are presented in Table 1.

## 3. Key Pre-Operative Advancements

Pre-operative optimisation of patients, an essential aspect of ERAS protocols, allows the patient to prepare both physiologically and psychologically for surgery. Pre-operative interventions that have been studied include a reduced fasting period, carbohydrate loading and various forms of counselling.

### 3.1. Carbohydrate Loading (CL)

Carbohydrate loading (CL) through pre-operative solutions containing complex carbohydrates such as maltodextrin is recommended by the ERAS Society as well as the European Society of Anaesthesiology [24,25]. The American Society of Anesthesiologists and the Canadian Anesthesiologists’ Society (CAS) also permit the consumption of clear liquids until 2 h prior to surgery, with the CAS going so far as to encourage it [24,25,26]. Fasting allows time for gastric emptying, thereby reducing the risk of intra-operative pulmonary aspiration [24,26]. The benefits of carbohydrate loading include metabolic optimisation, increased insulin sensitivity, reduced nausea and vomiting, reduced thirst, and reduced anxiety [24,25,26].

Pre-operative CL ensures that the body enters surgery in a fed state, which is preferable to the catabolic state that occurs in patients who fast for the standard 8 h pre-surgical period [24,25,26]. This catabolism results from the inhibition of insulin, which causes the release of glucagon and cortisol; however, CL has been shown to increase insulin sensitivity [24,25,26]. This negates these effects, reducing post-operative insulin resistance (mean increase in glucose infusion rate of 0.76 mg/kg/min), preserving glycogen and shortening LOS by 0.30 days compared with fasting [27,28]. CL also decreases the incidence of post-operative nausea and vomiting, as identified by Yilmaz et al., who reported significantly lower verbal descriptive scale scores (a measure of nausea) and antiemetic consumptions in carbohydrate-loaded patients following elective laparoscopic cholecystectomy [29]. CL has also been noted to reduce thirst, hunger, anxiety and malaise as well as increasing fitness when compared with patients who fasted from midnight the evening before surgery [30].

Despite these effects, CL appears to have no significant impact on rates of post-surgical complication [25]. CL also has yet to be researched in specific subspecialties, as conclusions are currently reliant on minimal data from studies in a small range of surgeries. Finally, the effect of CL in diabetic populations remains unclear, though experts feel it should be avoided due to its effects on insulin sensitivity [25].

### 3.2. Mechanical Bowel Preparation (MBP)

The role of MBP prior to elective colorectal surgery is well studied but controversial, owing to contradictory data from two reviews [6]. In 2009, Nelson et al. compared the efficacy of oral antibiotics (OA) in combination with MBP, intravenous (IV) antibiotics in combination with MBP, and OA and IV antibiotics together in combination with MBP [31]. They found that combined OA and IV antibiotics with MBP significantly reduced surgical wound infection compared to all other groups [31]. In 2011, Guenaga et al. assessed the need for MBP, judging that it was unnecessary as there was no significant difference in complication rates in the ‘no MBP group’ (*n* = 415) in comparison to ‘MBP group’ (*n* = 431) [32]. This led to confusion as there was no existing data regarding the use of OA and IV antibiotics without MBP; however, it was also clear that MBP was not necessary in colorectal surgery.

Subsequent trials in 2012 and 2015 found that OA plus MBP reduces the incidence of surgical wound infection by 40–57% (when compared with no OA or MBP), as well as reducing complications such as ileus and anastomotic leak [33,34]. Settling the issue, the American Society for Enhanced Recovery (ASER) and the Perioperative Quality Initiative (PQI) released a joint consensus statement in 2017 [6]. This statement included three recommendations for pre-operative MBP. These were as follows:

‘We recommend routine use of a combined isosmotic MBP with OA before elective colorectal surgery’.

‘We do not recommend use of MBP without concurrent oral antibiotics before elective colorectal surgery’.

‘We recommend against the use of hyperosmotic bowel prep solutions before elective colorectal surgery’.

The final recommendation concerns the use of hyperosmotic bowel preparation solutions. The reason for this is that hyperosmotic solutions cause changes in plasma osmolality as well as phosphate, urea, calcium and potassium concentrations and can lead to renal impairment [35].

### 3.3. Patient Education and Counselling

It is widely accepted that pre-operative patient education and counselling should be included in many if not all ERAS pathways. This allows the patient to manage their expectations before undergoing surgery [5], helps them to prepare psychologically, and can increase compliance to ensure a quick recovery. Forsmo et al. reported an average reduction in LOS of three days in colorectal surgical patients managed under an enhanced recovery pathway with a particular focus on counselling [36]. This research group also found that pre-operative stoma education reduces LOS without increasing the rate of readmission or early stoma-related complications [37], corroborating the findings of Younis et al. in 2012 [38]. This may be attributed to education on ‘independent stoma management’, which was identified as a limiting factor in the speedy recovery and discharge of patients [38].

Education and counselling have also been shown to improve pain control, especially in patients experiencing high levels of anxiety related to their surgery [19]. This effect appears to be consistent with all forms of patient education, from informal spoken information to leaflets [5], with one study advocating journaling on the grounds that it increases patient empowerment [39]. While the benefits seem clear, there is some evidence that providing excessive information to patients may actually reduce post-operative satisfaction. Barlesi et al. found that patients receiving both oral and written information were significantly dissatisfied following surgery in comparison to those receiving only oral information [40]. Overall, the ERAS Society and European Society of Thoracic Surgeons strongly recommend the inclusion of patient education and counselling in ERAS pathways, despite reporting ‘low’ levels of evidence [5].

## 4. Key Intra-Operative Advancements

Optimisation of intra-operative care is essential to avoid unnecessary intra- and post-surgical complications, which can have lasting medical effects as well as increasing LOS and reducing patient satisfaction. Important progress has been made in fluid management, antibiotic prophylaxis, minimally invasive surgery and intra-operative warming, to name a few.

### Fluid Management

Goal-directed fluid therapy (GDFT) is used in perioperative care to optimise oxygen delivery to end-organs. This is because evidence suggests that excess fluid resuscitation increases rates of complications such as pulmonary oedema, delaying patient recovery, while inadequate resuscitation may lead to complications such as pre-renal acute tubular necrosis [24]. GDFT may reduce the incidence of post-surgical complications and decrease LOS by 39%, as evidenced by Sinclair et al. in a population of 40 patients undergoing repair of a proximal femoral fracture [26,41,42]. It may also significantly reduce mortality following major surgery [43]. Despite this, GDFT has not yet been proven to be significantly more efficacious than restrictive or liberal fluid therapy, though ongoing research in the form of the Optimisation of Cardiovascular Management to Improve Surgical Outcome (OPTIMISE) II trial aims to address this gap in the literature.

Liberal fluid therapy has been shown to be harmful to patients undergoing major surgery. Gustafsson et al. found that post-operative complications (particularly cardiovascular) in 943 colorectal surgical patients increased by 32% for every additional litre of fluid given [44]. They also found that incidence of post-operative symptoms, which delay recovery, increased by 16% for each additional litre of fluid [44]. A number of studies corroborate these results, finding that intra-operative over-administration of fluids results in increased LOS [45]. This evidence led to the suggestion that restrictive rather than liberal fluid therapy should be used in ERAS protocols. However, the Restrictive versus Liberal Fluid Therapy for Major Abdominal Surgery (RELIEF) trial compared restrictive and liberal fluid administration in 2983 patients with the primary outcome of disability-free survival at one year post-surgery [46]. They found no significant difference in one-year disability-free survival between groups but did identify a significant 3.6% increase in incidence of acute kidney injury in the restrictive group [46]. This evidence suggests that restrictive therapy is equally, if not more, harmful than liberal therapy. Despite this, it remains to be seen whether GDFT is preferable to both liberal and restrictive therapies, though Bellamy’s theoretical U-shaped curve suggests that hypo- and hyper-volaemia are equally detrimental to a patient’s health [24], indicating that GDFT (which targets euvolaemia) is the best available practice.

Regarding research into GDFT, the OPTIMISE trial was the first multicentre trial to compare it to standard fluid management (*n* = 734); however, no significant reductions were found in rate of complications or 30-day mortality with the use of GDFT [47]. However, this study was underpowered and did report reduced mortality in the GDFT group, though it was not statistically significant (*p* = 0.07) [47]. Subsequently, the OPTIMISE II trial will aim address the same research question in a larger population (*n* = 2502) [7], though results are yet to be published.

## 5. Key Post-Operative Advancements

The post-operative phase is the most heavily researched in surgical perioperative care, with the National Institute for Health and Care Excellence (NICE) identifying 342 post-operative enhanced recovery studies in comparison with 123 intra-operative and 150 pre-operative [48]. They identified common components of these studies as early mobilisation, early introduction of diet, absence/early removal of nasogastric tube, absence/early removal of surgical drains and restricted fluid regimens [48]. These interventions are essential to fast-track patient recovery, reducing LOS and cost and improving patient satisfaction.

### 5.1. Early Mobilisation (EM)

EM is an essential aspect of ERAS pathways, with the ERAS Society recommending its implementation in elective colonic, rectal/pelvic and gynaecologic/oncologic surgeries [49]. The rationale underlying EM is that it reduces respiratory and thromboembolic post-surgical complications, which are associated with bedrest [49]. Enhanced recovery pathways implementing EM have been shown to reduce LOS by an average of 3.09 days following emergency abdominal surgery [8] and 4 days following pancreatic cancer resections [50].

Despite these benefits, adherence can be an issue in implementing EM, as demonstrated by Grass et al., who found that 58% of patients (*n* = 1170) fail to mobilise on post-operative day 1 [51]. This study also identified a significant relationship between failure to mobilise early and post-operative complications [51]. Major complications were increased by 9% in the delayed mobilisation group, and respiratory complications were increased by 8% [51]. Compliance with EM procedures has also been identified as an independent factor correlating to positive outcomes in laparoscopic colorectal surgeries [52]. Failure to comply with EM protocols is also associated with poor adherence to the remainder of the ERAS pathway [52]. This is particularly concerning as data show that patients with >80% adherence experience fewer complications and reduced mortality compared with less compliant individuals [50]. While these findings suggest that EM, although important, may be unobtainable in a large portion of patients, Fiore et al. found that adherence to EM protocols could be increased through ‘facilitated mobilisation’, whereby staff were specifically assigned to aid EM [53]. They also reported significantly increased step counts on post-operative days 1 and 2 [53]. This is encouraging, though staffing realities may limit the possibility for facilitated mobilisation in many healthcare systems.

### 5.2. Early Enteral Nutrition (EN)

Following major surgery, it was previously believed that initiation of post-operative nutrition should be slow and progressive, starting with clear fluids and working towards solids [54]. Evidence now clearly suggests that EN through oral or nasogastric feeding should be initiated as early as possible after surgery, with the European Society for Clinical Nutrition and Metabolism (ESPEN) strongly recommending (90% agreement) uninterrupted oral post-surgical nutrition [55] and a joint consensus statement from the American Society for Enhanced Recovery and the Perioperative Quality Initiative affirming its safety and efficacy [56]. Data show that initiation of EN during post-operative day 1 may be possible in up to 90% of patients [57]. Regarding evidence for the use of early EN, a 2016 meta-analysis comprising 15 studies and a total of 2112 patients found that early oral feeding reduces LOS by 1.44 days following upper GI surgery, without significantly increasing risk of common complications such as pneumonia and anastomotic leak [9]. Furthermore, early EN has been associated with significant decreases in LOS, total cost of hospitalisation and reduction in post-surgical complications [56]. Early EN has also been studied as a feature of ERAS protocols, with data showing reductions in overall morbidity and decrease in LOS by 2.28 days without causing further readmissions [58]. No significant relationship between early nutrition and rates of post-surgical complication was identified [58]. These data are taken from patients undergoing colorectal surgery, so it remains to be seen whether early EN is efficacious in other surgical subspecialties. The main pre-, intra- and post-operative advancements in ERAS are presented in Table 2.

## 6. Enhanced Recovery in Transplantation Surgery

Enhanced recovery is a relatively unexplored concept in the field of solid-organ transplantation when compared with many other subspecialties, such as orthopaedic and cardiac surgery. One possible explanation for this is a resistance to changes that may lead to a greater rate of patient and graft loss in the short term before successful new protocols are developed and ultimately improve patient outcomes [59]. Another is a resistance to practises that ‘fast-track’ surgical recovery, as this may be seen as a detriment to the patient-centred approach, which is fundamental to modern healthcare. The perception that ERAS protocols are rushing patient care, however, is certainly not the case as ERAS has been proven to reduce mortality and complication rates as well as bring down the overall costs of care. Finally, despite the cost-effectiveness of enhanced recovery, there may be financial disincentives to providing fast-track perioperative care for certain healthcare professionals [60]. A 2018 commentary on post-operative care in liver transplant recipients suggests that US anaesthesiologists may be dissuaded from certain fast-track protocols on the basis that patients who bypass intensive care may require prolonged treatment in post-anaesthesia care units [60]. Anaesthesiologists are not routinely able to charge for this care, so they may look negatively upon suggested changes in protocol [59,60]. This financial disincentive is specific to privatised healthcare models such as the Bismarck, national health insurance and out-of-pocket models [61], in which care providers may be influenced in their actions by the associated costs.

### 6.1. Liver Transplantation

The first indications of perioperative care resembling ERAS protocols in liver transplantation (LT) discussed the idea of early post-operative extubation. The earliest report was published in 1990 in a conference paper reviewing extubation of LT patients either immediately or within 8 h post-surgically [62]. However, this was observational, and no specific protocols were in place to guide extubation practices. In 1997, Mandell et al. used retrospective analysis of extubation practices to formulate a protocol, which they used to determine time of extubation in 67 patients [63]. Of these, 16 were extubated immediately following surgery and none required re-intubation [63]. The study also reported an average reduction in cost of $2709 associated with immediate extubation; they concluded that this was both safe and cost-effective [63]. This, along with another 1997 study that reported the safety of rapid extubation but found no significant reduction in LOS [64], was the first use of ERAS-type procedures in solid-organ transplantation. Since 1997, further studies have corroborated these findings of cost-effectiveness of early extubation following LT, with Taner et al. reporting cost reductions related to a decline in ICU requirement [65]. Their protocol dictated that 60.1% of 523 patients did not require post-surgical ICU admission, and they reported a failure rate (indicated by subsequent ICU requirement) of just 1.9% [65]. Later studies supported the bypass of ICU following LT, with Mandell et al. also identifying a reduction in overall LOS [66]. Early extubation has also been associated with reductions in complication rates following LT, specifically sedation-delirium and pneumonia (problems associated with mechanical ventilation) [59]. A possible explanation for these results is that early extubation allows the patient to avoid the deleterious effects of mechanical ventilation, though the topic of post-LT ventilation is debated, with some arguing that positive end-expiratory pressure causes backflow of blood into the newly transplanted liver and others reporting no effect on graft function [67].

Owing to these advancements in early extubation, research has been carried out into full ERAS-type perioperative protocols in LT, with considerations such as fluid restriction, use of intra-operative sedatives and evaluation of anaesthetic agents [59,67]. Biancofiore et al. also implemented a continuous quality improvement programme, which allowed for a ‘learning curve’ [68]. With this, they saw the proportion of patients receiving immediate post-operative extubation increase from 19.0% to 82.5% over a five-year study period [68]. These developments clearly show the beginnings of ERAS in LT; however, recent years have seen a loss of traction, possibly due to changes in US organ allocation practices in 2002 [10]. New laws incorporate the Model for End-Stage Liver Disease (MELD) score into organ prioritisation; this is a prognostic tool that predicts three-month survival in prospective LT patients [10]. This addition disincentivises pre-surgical optimisation of the patient, which may see them deprioritised, causing them to miss out on life-saving organ transplantation. Optimisation is a key aspect of enhanced recovery, and therefore, implementation of the MELD score may be seen as a precipitating factor in the decline in progress of ERAS in the field of LT. It is imperative that the field of transplantation does not fall behind in the development of perioperative care but instead begins to incorporate these new transplantation criteria into ERAS protocols. Brustia et al. have begun this process, incorporating a cut-off MELD score of 25 into their study of a 26-point ERAS protocol in LT patients [11]. They reported a 47% reduction in LOS in patients in the ERAS group when compared with controls, with no significant difference in the rate of complications [11]. This pilot study evidences the potential for enhanced recovery protocols to improve patient outcomes in spite of restrictive new transplantation laws, though just 10 patients were managed under the ERAS protocol, and further, larger studies are required to corroborate these results.

### 6.2. Renal Transplantation

The uptake of ERAS into the field of renal transplantation has been equally slow. In 2019, Morkane et al. concluded that there was a high degree of heterogeneity between perioperative practises across 23 renal transplantation (RT) centres [69]. They found that 27.3% of centres utilised cardiac output measures to guide fluid administration, 40.9% aimed for specific intra-operative targets of central venous pressure, and 54.5% use fentanyl-based patient-controlled anaesthesia alongside transversus abdominis plane block [69]. This highlights the poor standardisation of perioperative care with regards to RT, despite evidence that enhanced recovery reduces length of hospital stay and confers better pain control than standard practices [70].

In recent years, multiple studies have assessed the feasibility and efficacy of ERAS protocols for patients undergoing RT. In 2016, Kruszyna et al. assessed the feasibility of an ERAS protocol in 45 deceased-donor kidney transplant recipients in a single-centre case series [12]. They found that median LOS was 10 days and that three-month graft survival was 97.8%, but also reported a serious complication rate of 6.6% and unplanned readmission rate of 8.9% [12]. They determined that ERAS was feasible in RT and that further improvements could be made with the implementation of financial policies by healthcare regulators [12]. Following this, Halawa et al. compared outcomes between a cohort of 135 patients whose perioperative care followed an ERAS programme and 151 patients receiving traditional care [71]. This ERAS programme included aspects such as goal-directed fluid management, early discharge planning and pre-operative carbohydrate loading. The study noted significant reduction in LOS in the ERAS group in recipients of both deceased- and live-donor transplants. They reported a coincident reduction in total cost of care by £2160 in living-donor recipients and £3078 in deceased-donor recipients [71]. Post-operative morphine requirement was also reduced in the ERAS cohort. Dias et al. performed a prospective study of 200 patients, of which 100 were treated in accordance with a standardised ERAS protocol, and found similar results with respect to reduction in LOS [72]. Interestingly, they also reported a significant reduction in the incidence of delayed graft function in patients receiving deceased-donor transplants treated under the ERAS protocol [72].

Many of the recent advancements in RT focus on the potential for ERAS protocols to reduce pain perception and opioid requirements following surgery. Most notably, Campsen et al. assessed the effectiveness of an ERAS protocol that used pregabalin in pre-operative care to desensitise the patient’s nerves, before using ketorolac (an NSAID) intra- and post-operatively [13]. This study aimed to reduce perioperative narcotic use without increasing complication rates and negatively impacting on the standard of care. In patients receiving the non-opioid analgesic regimen, there was a 40% reduction in morphine dose equivalents, a measure of cumulative narcotic use throughout hospitalisation [13]. No significant difference in complication rates was identified between study cohorts [13]. This is the only study to-date in which a pain-focused ERAS protocol was implemented in RT patients; however, various studies have addressed the problem of chronic post-surgical pain (CPSP) in this population. These studies have found that 24.6% to 33% of donors experience some degree of CPSP for at least 19 months after surgery and identified the presence of pre-operative and acute post-operative pain as risk factors for its development [73,74]. That perioperative pain before or after RT is a risk factor for development for CPSP [73,74] shows the importance of comprehensive analgesic guidelines and standardised practices such as ERAS protocols to ensure patient comfort in the long term [75]. To this end, an ongoing study at the Thomas Jefferson University aims to further address perioperative non-opioid analgesia as an aspect of ERAS protocols in RT.

### 6.3. Other Areas of Solid-Organ Transplantation

In addition to LT and RT, pancreatic, cardiac, pulmonary and intestinal transplantations are now common. The ERAS Society has published consensus guidelines for the implementation of enhanced recovery in lung [5], cardiac [76] and colorectal [77] surgeries; however, these guidelines do not include transplantations. They have also produced guidance on perioperative care for pancreaticoduodenectomy [78] but are yet to address pancreatic transplantation. Very little research has been conducted into the use of ERAS in these other forms of solid-organ transplantation, though a feasibility study was carried out regarding enhanced recovery and opioid-sparing analgesia in lung transplant recipients [79]. The study by Lewis et al. included 48 patients and concluded that enhanced recovery and opioid-sparing protocols achieve acceptable pain management, with just three patients requiring opioid analgesia at discharge [79]. While this may indicate the beginnings of ERAS in pulmonary transplantation, there is a long way to go before it becomes commonplace. Further research should begin to assess the efficacy of ERAS pathways in pulmonary transplantation in comparison to standard perioperative care and their feasibility in cardiac, intestinal and pancreatic transplantation. The main ERAS developments in transplantation surgery are presented in Table 3.

## 7. Conclusions

ERAS pathways offer safe and cost-effective approaches to perioperative care, which improve patient outcomes without increasing rates of complication. Since the inception of ERAS in 1997, so-called ‘fast-track’ surgical pathways have become widely used in multiple specialties, and the standard of perioperative care has improved substantially, in no small part due to the work of the ERAS Society. Key advancements have been made, including pre-operative carbohydrate loading, patient education, GDFT and early enteral nutrition. However, the uptake of ERAS in transplantation surgeries has been slow, leading to a paucity of literature in the field. While recent years have seen some developments in ERAS relating to liver and kidney transplants, other areas of solid-organ transplantation—including lung, heart and pancreas transplantation—are yet to make notable progress. Future research should address the feasibility and efficacy of ERAS in these areas, and emphasis should be placed on the speedy incorporation of ERAS pathways into standard perioperative care for transplant surgeries.

## Figures and Tables

**Table 1:** Summary of key events in the history of ERAS.

Advancement in Enhanced Recovery	Year
Kehlet publishes paper introducing the concept of ‘fast track surgery’ [3].	1997
Kehlet publishes first paper showing efficacy of ERAS in sigmoid resection [14].	1999
ERAS Study Group is formed.	2001
Study shows that perioperative care is not consistent across Europe [17].	2005
ERAS Study Group publish first consensus guidelines for colorectal surgery [19].	2005
ERAS Society is formed.	2010
Meta-analysis shows efficacy of ERAS [20].	2010
Study confirms findings that perioperative care is inconsistent across Europe [18].	2012
Study assesses possibility of large-scale implementation of ERAS protocols [21].	2013

**Table 2:** Summary of key advancements discussed.

Advancement	Summary of Key Points
Carbohydrate Loading	∙ Aims to avoid pulmonary aspiration by allowing gastric emptying [24,26] ∙ Increases insulin sensitivity [24,25,26] ∙ Reduces LOS by 0.3 days [27,28] ∙ No significant impact on complications [25]
Mechanical Bowel Preparation	∙ MBP plus OA and IV antibiotics confers largest reduction in surgical wound infection [31] ∙ No significant reduction complications with singular use of MBP [32] ∙ MBP plus OA reduces surgical wound infection and complication rates [33,34]
Patient Education and Counselling	∙ Reduces LOS by 3 days [36] ∙ Improves pain control [19] ∙ Information overload may reduce patient satisfaction [40]
Goal-Directed Fluid Therapy	∙ Reduces LOS, complications and mortality [24,26,41,42,43] ∙ Not yet proven significantly better than restrictive fluid administration ∙ OPTIMISE II study hopes to address this [7]
Early Mobilisation	∙ Reduces LOS by 3–4 days [8,50] ∙ Compliance with EM protocols can be improved with ‘facilitated mobilisation’ [53] ∙ Despite benefits, EM may not be feasible for reasons including inadequate staffing
Early Enteral Nutrition	∙ Reduces LOS by 2.28 days following colorectal surgery [58] ∙ May reduce complications [56] ∙ Requires investigation in a broader range of surgical subspecialties

**Table 3:** Summary of ERAS developments in transplantation.

Type of Transplantation	Key Developments
Liver	∙ Early extubation reduces LOS and healthcare costs [63,64,65] ∙ Many patients do not need to be cared for in ICU, and avoiding this can significantly reduce healthcare costs [65,66] ∙ Changes to US organ allocation that incorporate the MELD score may disincentivise patient optimisation [10] ∙ MELD score cut-offs can be used to determine which patients enter the ERAS pathway and which receive standard perioperative care [11]
Kidney	∙ ERAS pathways are feasible and reduce LOS following renal transplantation [12,71] ∙ ERAS protocols reduce healthcare costs by £2160 in living-donor recipients and £3078 in deceased-donor recipients [71] ∙ ERAS significantly reduces delayed graft function following deceased-donor transplants [72] ∙ Pregabalin-ketorolac (non-opioid) regimens reduce analgesic requirements (given as morphine dose equivalents) by 40% [13]
Lung	∙ ERAS pathways with opioid-sparing analgesia offer acceptable pain control in lung transplant recipients [79]

## Data Availability

No new data were created or analysed in this study. Data sharing is not applicable to this article.
